# Beyond inclusion: Enacting team equity in precision medicine research

**DOI:** 10.1371/journal.pone.0263750

**Published:** 2022-02-07

**Authors:** Melanie Jeske, Emily Vasquez, Stephanie M. Fullerton, Aliya Saperstein, Michael Bentz, Nicole Foti, Janet K. Shim, Sandra Soo-Jin Lee

**Affiliations:** 1 Department of Social and Behavioral Sciences, University of California, San Francisco, California, United States of America; 2 Department of Sociology, University of Illinois, Chicago, Illinois, United States of America; 3 Department of Bioethics & Humanities, School of Medicine, University of Washington, Seattle, Washington, United States of America; 4 Department of Sociology, Stanford University, Stanford, California, United States of America; 5 Division of Ethics, Department of Medical Humanities and Ethics, Columbia University, New York, NY, United States of America; University of Toronto, CANADA

## Abstract

**Purpose:**

To identify meanings of and challenges to enacting equitable diversification of genomics research, and specifically precision medicine research (PMR), teams.

**Methods:**

We conducted in-depth interviews with 102 individuals involved in three U.S.-based precision medicine research consortia and conducted over 400 observation hours of their working group meetings, consortium-wide meetings, and conference presentations. We also reviewed published reports on genomic workforce diversity (WFD), particularly those relevant to the PMR community.

**Results:**

Our study finds that many PMR teams encounter challenges as they strive to achieve equitable diversification on scientific teams. Interviewees articulated that underrepresented team members were often hired to increase the study’s capacity to recruit diverse research participants, but are limited to on-the-ground staff positions with little influence over study design. We find existing hierarchies and power structures in the academic research ecosystem compound challenges for equitable diversification.

**Conclusion:**

Our results suggest that meaningful diversification of PMR teams will only be possible when team equity is prioritized as a core value in academic research communities.

## Introduction

There have been resounding calls for increased workforce diversity (WFD) and greater inclusion of underrepresented researchers in genomics and academic science more broadly. Growing awareness of systemic racism, in the wake of ongoing racial disparities in the COVID-19 pandemic and continued extrajudicial killings by police, has prompted scientific communities to elevate conversations about racism, diversity, equity, and inclusion. Indeed, across the US, myriad academic institutions, funding agencies, and scientific journals have released statements on systemic racism and launched diversity and inclusion initiatives that seek to address longstanding disparities and institutional harms [1–5]. Since 2018 our study team has engaged in an ongoing study of efforts to diversify participation in precision medicine research (PMR); during this time, these major social and political events in the US created opportunities for us to learn about the challenges of diversifying the scientific teams themselves. Although the focus of our broader investigation is on diversity in research participation, WFD emerged as a critical dimension. In this article, we focus on challenges that genomics and precision medicine research teams face in not only diversifying their membership and composition, but also in having that diversity manifest in inclusion that is meaningful and equitable.

Often not explicitly defined, “workforce diversity” typically refers to racial and ethnic groups underrepresented in the scientific workforce (e.g., Black, Indigenous, people of color) and also includes women, sexual and gender minorities, individuals with disabilities, and those from socioeconomically disadvantaged groups [6]. Reflecting the terms through which our participants framed WFD, findings reported here focus primarily on racial and ethnic diversity. Broadly, WFD is purported to bring myriad benefits to team science: research shows underrepresented investigators produce novel research [7], generate more innovative solutions to problems, and publish more influential scientific papers [8]. Given this evidence, multiple funding agencies now emphasize that science can only reach its fullest potential when it can recruit and retain a diverse workforce. In response, NIH, the major funder of biomedical research in the US, has funded several initiatives aimed at increasing WFD diversity, including the UNITE initiative launched in February 2021 to focus on structural racism in the biomedical workforce and to promote diversity and inclusion across the biomedical research enterprise [9, 10].

Yet WFD discussions often do not emphasize *equitable* diversification: while WFD initiatives focus on increasing underrepresented researchers, conversations about power and inequity on teams remain peripheral. This paper highlights why equity on research teams must be a central feature of WFD initiatives. Drawing on existing frameworks [11, 12], we define equity in scientific research as the absence of disparities in opportunities, leadership positions, and access to academic networks and resources that are systematically associated with social position, especially, but not only racial and ethnic identity.

A broad literature demonstrates systemic racial and ethnic stratification within the academic research ecosystem. Investigators of color face challenges to entry at all levels of scientific training; once in academic science, investigators of color are more likely to be saddled with service work that is undervalued in promotion and tenure review, and they earn less than their white colleagues [13–18]. Building on this scholarship, we identify key challenges to equitable diversification of precision medicine research (PMR) teams. PMR is an important site to investigate this issue, because it is a site of multidisciplinary collaboration and has increasing emphasis on the recruitment of diverse participants to diversify genomics databases. Diversification of research participants is inextricable from issues related to diversification of the research workforce and who and how the research is implemented. Our findings point to necessary reconfigurations to scientific research infrastructures to achieve equity.

## Materials/Method

Data reported are drawn from a large, multi-sited qualitative project that investigates how commitments to diversity and inclusion are interpreted and operationalized in five PMR studies across three national consortia. The three NIH PMR consortia were selected because of their geographic diversity, federal funding, and their expressed commitment to recruiting and engaging diverse participant populations. Across the three consortia, five individual studies were selected because they were located in geographically diverse areas, and used heterogeneous strategies to engage, recruit, and retain underrepresented individuals as research participants. Institutional review board approval was obtained from the University of California, San Francisco and Columbia University.

This study utilized multiple qualitative methods, including in-depth interviews, observations, and document review. Interviews (60–90 minutes) were conducted with 102 purposively-recruited investigators, research staff, NIH program officers, and community advisory board members. Demographics of our sample are provided in Table 1. Interviews were conducted via Zoom or in-person, audio-recorded, and transcribed. Interviews followed a semi-structured, open-ended format. During these interviews, we principally explored the engagement, recruitment, and retention of underrepresented participants, as well as how study teams were operationalizing diversity mandates in their research. Interview guides were tailored according to investigator and staff member roles, to ensure that questions were appropriate for each given participant. Observations focused on study team and consortium meetings and scientific conferences, totaling over 400 hours. Detailed fieldnotes were taken during observations.

**Table 1:** Sample demographics.

Gender	Number	Percent*
Women	65	64%
Men	37	36%
Nonbinary or Other	0	
**Race**		
White	72	71%
Black or African American	16	16%
American Indian and Alaska Native	0	
Asian	6	6%
Native Hawaiian and Other Pacific Islander	1	1%
Two or more races	3	3%
Not reported	4	4%
**Ethnicity**		
Hispanic/Latino	9	9%
Not Hispanic/Latino	92	90%
Not reported	1	1%
**Age**		
18–65	93	91%
65+	9	9%
**Total**	**102**	

*Percentage rounded to nearest one percent.

All data, including interview transcripts and observation fieldnotes, were coded in Dedoose. Using a modified grounded theory approach, we developed a codebook based on a priori concepts and inductive coding [19]. To maximize intra- and inter-coder reliability, our team periodically jointly coded the same data and discrepancies were discussed and reconciled. Following coding, the research team wrote analytic memos on the codes and their corresponding data, and these were discussed in team meetings.

Because issues of WFD were raised by our study participants throughout our interviews and observations, we added questions about workforce diversity to our interview guides, and we coded and analyzed data on this emergent theme. We consider theoretical saturation to have been reached on our findings on WFD, as we found that additional data collection no longer yielded new empirical dimensions or conceptual insights related to the relationships among workforce diversity, equity, and inclusion. In this article, we foreground interview data with investigators, research staff, and funders because they contained the most detailed reflections about the challenges of WFD and equitable inclusion of research team members.

Institutional Review Board approval was obtained from the University of California, San Francisco and Columbia University. All institutions involved in human participant research received local IRB approval. Informed consent was obtained from all participants as required by IRBs.

## Findings

Our analysis shows that (1) underrepresented individuals report they are selectively and disproportionately hired into frontline research staff positions, are often viewed as experts on diversity *only*, and perceive limited opportunities for upward mobility and contributing other dimensions of their expertise. Interviewees articulated how (2) existing hierarchies and power structures compound challenges to the equitable diversification of research teams. Finally, interviewees suggest that (3) equity requires building an infrastructure for mentorship, opportunity, and meaningful inclusion.

### 1. Underrepresented team members relegated to frontline roles and diversity concerns

Perceived as a key element for successful recruitment of diverse cohorts, increasing diversity among research staff has become a ubiquitous strategy in PMR [20]. In most studies we observed, study team diversity was most prominently discussed regarding “on-the-ground” or frontline staff, such as research coordinators, recruiters, and translators. For studies that increasingly seek diverse cohorts, investigators recognize the importance of team members who “look like,” and whose experience may resonate with, potential participants. One investigator explained that their team explicitly tried to hire diverse frontline staff, and highlighted that this was a key strength of their study:

[We] have made a point of hiring staff, especially for frontline staff who are going to be out there interacting with participants… who are familiar with those communities that we’re trying to engage. And often that means they themselves might identify as being part of that community or, in some way, they have had existing relationships and knowledge and involvement in some of these communities.

As another researcher observed about potential participants, “I think that their ability to say, ‘You know, I look like you, I signed up for this study,’ makes a huge difference.”

PMR investigators spoke openly about their own realizations of the importance of WFD in their research teams and studies. One investigator explained this issue was not on her radar until her primarily white team attempted to conduct a community engagement event with an African American audience: “I realized sitting there…the level of distrust…was through the roof, compared to the other groups that we had done… . I was like, ‘Wow, we did not think this through very well.’” For their part, frontline staff members also recognized the importance of connecting with their own communities. As one research coordinator related, diversity among staff has “really helped because…when participants come in, I think that makes a real difference to see somebody from their own ‘community,’ quote-unquote, and it’s helped us in terms of our recruitment.” A genetic counselor explained it was important to have someone who shared participants’ “culture” to accompany them throughout the study:

That person is involved in their pretest stuff, involved in their return of results, and involved in their follow-ups so there’s some continuity and is someone who is from their culture, as well, who has that background and can support them…. Maybe instead of me being like, "I need to learn everything and respond to every culture," maybe bring somebody else in who already does.

Participants also underscored that this approach can be problematic, as it assumes one person can represent the ideas, values, and experience of an entire group. As one study staff member explained, she would like to see more underrepresented staff and leadership “so it’s not one person bearing the burden of making sure the concerns of an entire non-monolithic population are heard, because that is a lot to put on one person.” This mirrored concerns raised by underrepresented team members who said that because they “look like” the populations being recruited, study leadership expected them to speak on behalf of those groups, perpetuating harmful stereotypes.

Indeed, insofar as diverse team members were understood to be important because they helped with the recruitment and retention of diverse participants, this reflected a largely instrumental justification for the goal of WFD. A focus on the benefits of a diverse frontline staff for participant recruitment also served to deflect attention on the lack of diversity elsewhere in the precision medicine research enterprise. For example, one investigator reflected candidly that while “at least on face value, we’re doing pretty well with at least bringing diverse participants in the door,” she acknowledged that “we are doing crappy at diversity within respect to consortium staff. We’re doing pretty darn crappy when it comes to diversity with respect to users of the data.” Another respondent stressed that it was necessary to:

Diversify the biomedical research workforce that will use our data…. Otherwise [it’s] the usual suspects: R01 universities, and we already know the biomedical workforce by the statistics…about who gets grants and who doesn’t get grants, and who’s successful winning awards…. We have largely seen [an] absence of inclusion of underrepresented and minority and other groups in the biomedical research workforce.

Additionally, underrepresented team members working in PMR reported difficulty being recognized for their contributions apart from diversity work. As one study-staff member explained, there is an “expectation that that’s the only research that I do. It just so happens that it’s the research that I’m interested in, [but this] is not the case of everyone.” In addition to the limitations this creates for underrepresented team members to contribute more broadly to the science, this expectation also seemed to release others on the team from responsibility for meaningfully engaging with the difficulties of research across racial, ethnic, language or socioeconomic difference. One study staff member explained:

Every Brown, Black person feels this way. They’re like, “we want to talk about anti-racism efforts,” [and they] look to the one Black person in the room. Why is it our burden? We already have a burden to be in this field…just to be Black and Brown and in this country, but in this field, especially in genetics, [it is] all white people, overwhelmingly clueless, white people.

Such tokenism translates into limited power to shape the research agenda, study design, and deliverables on a given study. As another staff member explained, it is important for underrepresented individuals to be there “not as a token, or to meet that checkmark, but to actually let them have feedback without feeling that my job will be in jeopardy.”

While many investigators spoke about the need for diversity among frontline staff, few explicitly discussed diversifying study leadership. More frequently, recognition of the need to improve WFD on study teams we observed led to discussions about solutions that would not displace current leadership. For example, in one meeting, an investigator noted that studies across their consortium had not done a satisfactory job involving diverse research teams and investigators. In response, she proposed the team to consider recruiting underrepresented summer interns to work with consortium data. Reflecting on the lack of diversity in leadership roles in her consortium, another investigator explained that WFD among leadership cannot be an afterthought:

[Our] senior staff and even some of the team leads are overwhelmingly straight middle-aged white cis heterosexual. This is not the way. Yes, there’s something to be said for people who are sensitive to diverse perspectives, but I think that there’s nothing that can really stand in for lived experience… . I think we didn’t think about that part of our responsibility, and [now] we’re kind of stuck. And I don’t think we can use the cop-out, “Well, there weren’t any qualified candidates.” There are qualified candidates.

She related that her consortium recently tried to hire underrepresented candidates for leadership roles, but was unsuccessful in part because the study’s design and approach had already been defined without them. She argued it is crucial that underrepresented investigators assume leadership positions early in a study’s development to have real power in shaping the project.

When asked how scientific communities can assess whether WFD efforts have been successful, another investigator responded, “I would want to look at the organizational structure, who’s in different leads. How many principal investigators, for example? How many leads of the working groups are diverse?” Similarly, another investigator explained, “True diversity is when you start to see inclusion. You see the makeup of the leadership is more diverse, more representative of Black and Latinx leaders, more women in administrative positions.”

### 2. The academic research ecosystem perpetuates existing networks of power

Participants articulated how existing hierarchies of academic institutions and networks perpetuate inequalities, by rewarding investigators who are already well-networked and funded. While this practice may be unintentional, it is harmful in its impact. One program officer explained:

For example, ‘Oh, I know the person that trained this person, they have to be good’… There’s a reason that you keep hiring people who are non-diverse. If you keep getting it through the pipeline of the people you know, that’s not going to work. You may not know this person who trained that person but they still can have a very good training. You’re just not giving them the opportunity, because you’re just going with what you know.

As she put it, investigators often select collaborators who are familiar to them, not because they are necessarily better than others, but due to familiar social networks. An investigator elaborated,

Many of the opportunities that become available…have to do with who you know. If the people who are in charge of things only or mostly include the people that they know, they’re going to be including people that look like them. They have to intentionally reach out to people who do not look like them. [But] they have no real reason to do that…. I’m noticing that the mood in the country, as the country becomes more diverse, the people who are at the seat of power have become afraid that they’re going to lose their power. So they hold on to their little power structures.

Another research team member agreed, saying “they’re clinging to power and they’re clinging to influence and holding everyone else down.” Many of our participants described coming to greater recognition of their own role in perpetuating these dynamics, motivating efforts to change their own practices. One investigator acknowledged that previously, many of her interns came from her social networks and word of mouth, and described her growing awareness of how this unintentionally reproduced inequity, but also how this could be addressed with institutional interventions:

We’ve moved towards public posting. Everybody can apply and we select the best candidate instead of this word of mouth, because that’s been identified as a source of lack of diversity, this “you got to know someone to get there.” I didn’t even think about that myself, like a friend emails me, "Sure, I’ll take your son or your daughter into my lab and help them out." Well, that’s perpetuating the disparities, right? But we all have to be trained and taught. What’s helping me get interns that are diverse is that [my institution] has launched programs specifically that pull from applicants and then match people with labs. I can sign up and say, "I have room for three interns. If you can bring them in, I will take them." Then they create a program, so that there’s also common things where all those interns come together and share experiences like a whole program.

This investigator highlighted that expanding networks requires shifts to individual as well as organizational and structural practices. Yet another investigator, involved in efforts to increase data access, explained:

I see the vision for having sort of an ecosystem like this… . I see this with my colleagues in my own department, that not everyone has access to the data like I do. So opening up that access to other researchers who may have really clever ideas of how to analyze, I can see the value of that… . They aren’t funded, or they don’t have access, or work at a major university that’s part of [this], or have a [name of established, well-funded researcher] that has mentored you.

PMR investigators recognized the importance of networks and associated access to funding to success in the field, and how this stratifies opportunities for underrepresented investigators. Participants noted that this is not an individual-level issue; structural barriers reproduce inequities in the investigators and institutions who are routinely funded. As a program officer explained,

I think that a lot of [grant] reviewers are looking at where you’ve had the best success. You just don’t have time to wait for [institutions without a funding history] to get on board. I’ve worked with a number of consortia for which that’s the problem. And it’s not that they’re not willing and wanting, it’s just… a lot of people don’t have the clinical decision support and those kinds of things. Getting all of that worked out takes a while.

That is, grant reviewers are often concerned about institutional capacity to successfully achieve study goals. This concern is clearly legitimate in many cases, as reviewers are explicitly asked to assess the institutional environments and resources of grant applicants and their ability to implement their proposed study activities. However, this pattern combined with the general lack of funds to *build* institutional capacity for research, has the unintended consequence of contributing to the centralization of power and resources among well-funded institutions and, consequently, the composition of the researchers who tend to be based at those institutions. Even as some initiatives attempt to include sites without a track record of funding, these efforts do little to disrupt the balance of power and can be reduced to what one consortia leader described as “grantsmanship” strategy of bringing “less well-funded institutions to a seat at the table because they’re partnered with others who have more experience, more grantsmanship, more track records, stuff that does influence whether you get grant funded.” And yet, it remains unclear how this strategy manifests in equitable study leadership, budget authority, and decisions about what diverse team members end up doing in such studies.

### 3. Equity in science requires well-resourced infrastructure for mentorship, opportunity and meaningful inclusion

Successful approaches to meaningful inclusion mentioned by our participants included reliance on “diversity heroes,” or successful investigators who have prioritized mentorship of underrepresented junior investigators. One investigator described a well-connected underrepresented investigator who made training and mentoring investigators of color “a major focus” and leveraged personal networks toward achieving this goal. However, our participants reminded us that this responsibility and commitment must be shared more widely. That is, they noted that current practice relies too heavily on individual investigators who are, as one investigator put it, “committed to seeing African Americans and people of color succeed.” She explained that she and others continue this practice, but that concerted and systematic efforts to expand opportunities are needed:

We need to continue to have opportunities that are designed to bring people of color into scientific [communities], get them the skills that they need to be able to participate as scientists.

As this investigator suggests, the growth and sustainability of WFD cannot depend on the good will or ad hoc efforts of individual researchers to offer mentorship, but rather needs to be ensured through institutional commitments to change. Another investigator emphasized that the establishment of formal training programs for undergraduate and graduate students, as well as a funded network of working groups in which mentoring for diverse junior scientists could take place, set their study apart. She recognized this as “one of the core [study] goals,” funded in parallel with research advancement and community engagement. With regard to training students from the community the study is located in, a study-staff member estimated:

[We’ve] had something like 200 students, and there are any number of students now that are working in science and all kinds of areas. And we have probably three or four scholars, people who were participating as undergraduates who are now investigative staff and [study] investigators. That’s a testament to what can happen when you spend enough time and resources.

She emphasized that local Historically bBlack cColleges and uUniversities have been a focus in these kinds of WFD initiatives for years, and so the programs’ success only required that the study partner with these institutions and that NIH funding was available to support the collaboration. Further, once underrepresented professionals are engaged in the field, our interviewees emphasized that there must be continued commitment from academic and funding institutions to supporting their research programs. As one investigator put it, “You bring people to the table. That’s maybe diversity, but are they able to really work on the issues that they want to work on? Are they able to do that? And is the structure set up to be successful in that way?”

## Discussion

Our data suggest that WFD initiatives must explicitly address the social structures of science that engender inequity on PMR teams. Participants highlighted a need for structural change and a commitment to diversity that extends beyond tokenism or “checking the box.” Ultimately, they, and we, argue that WFD makes for better science, but only when *equity* is exercised as a core value of scientific teamwork.

### Instrumental diversity risks undermining goals of equitable inclusion

Participants described what we call *instrumental diversity*: that is, efforts that enlist underrepresented team members for instrumental goals (e.g. recruitment) but do not prioritize them in leadership roles or stages of the study lifecourse (e.g., conceptualizing study aims and design). Leveraged as on-the-ground staff who might more successfully connect with participants who “look like them” or as tokenized experts on underrepresented communities, instrumental diversity efforts further inequities in PMR teams and limits underrepresented team members from actively participating in knowledge making processes. Tokenism has also been shown to lead to higher depression, anxiety, and social exclusion [21, 22]. Underrepresented team members often perceive that they are valued for contributions on diversity matters, but can be excluded from contributing to study activities unrelated to diversity.

Instrumental diversity also suggests a numerical accounting for diversity on study teams, without acknowledging other salient features of team hierarchy. This approach fails to create inclusive environments in which underrepresented research team members feel safe, supported, and encouraged to contribute intellectually. The lack of full integration—where diverse team members are missing from leadership—recapitulates deeper inequities within the field, while touting diverse representation at a surface level [22, 23]. To combat this trend, underrepresented researchers must be prioritized for study leadership and from the earliest stages of study planning.

As predominantly frontline staff, underrepresented team members in our study explained that they frequently found themselves in the uncomfortable position of becoming spokespeople for the study. This is especially concerning when trust—and the trustworthiness of biomedical research and healthcare institutions—is fragile for many from underserved communities [6]. This approach fails to recognize the importance of WFD on equity grounds [24]. Instead, instrumental diversity can compound inequity by both boxing underrepresented staff members into such roles and then relying upon them to broker relationships with or act as bridges to underrepresented communities.

### Reliance on social networks perpetuates inequities in the research ecosystem

While training has been a focus for increasing diversity in the genomics and PMR workforce, participants explained resolving issues in the existing infrastructure is also crucial. As others have argued [25], our study underscores that recruiting a diverse workforce is insufficient when the very infrastructure of academic science—from funding mechanisms to training models—perpetuates inequity. Participants in our study echoed resounding calls for structural changes [18, 26] to address goals of diversity. We now turn to three components of scientific infrastructure where our findings suggest changes can promote more equitable diversification on PMR teams: (1) research funding, (2) training and mentoring models and (3) research study governance.

#### Diversifying research funding

Receiving grants does more than fund research; it affords prestige and social capital to researchers and thus profoundly shapes scientific careers [27]. Study participants discussed multiple ways in which the current funding structure perpetuates existing networks of power. Federal funding agencies, including the NIH, privilege investigators at high-profile research universities and those who are well-networked and previously funded, with recent estimates showing that 10% of NIH investigators receive over 40% of NIH funding [28]. Research shows racial disparities in R01 funding [29], and that African American investigators are more likely to submit awards on topics that receive lower funding, such as community and population health. Effectively, the funding structure disproportionately benefits already well-resourced investigators and those who choose topics perceived as “exciting” to the scientific community, which is disproportionately white [13]. This “usual suspects” approach, in which award decisions are based on investigators’ track record of successful grantsmanship, ensures the concentration of power among relatively few researchers. While investigators in our study recognized this and discussed workarounds, participants highlighted that this problem demands funder attention.

Our study reveals a similar reliance on existing networks and infrastructures. Researchers reported partnering with external sites that have some established biomedical research infrastructure, avoiding the time-consuming and costly need to build infrastructure de novo and train staff and healthcare providers. These nodes of “baked-in” diversity were perceived as prudent investments compared to the riskier proposition of community-based institutions with little or no track record or limited infrastructure. This further entrenches networks of power that stymie novel pathways for integrating diversity [30].

#### Restructuring training and mentoring for retention

Our data show that relying on “diversity heroes,” who champion WFD, is insufficient to fully integrate and sustain underrepresented trainees. Further, research shows that underrepresented trainees frequently are deterred from academic careers as their training progresses [31, 32], highlighting that recruitment alone is not enough. Our participants spoke to the need for explicit investment in training diverse researchers and creating inclusive mentoring environments as critical to achieving equity. Infrastructure is required that supports underrepresented trainees through career advancement. Given the importance of extant networks for professional development and training, our findings suggest that principal investigators must prioritize mentorship of future team members from underrepresented groups and wield their social capital toward such ends. To further these efforts, funders and academic institutions should provide sponsorship to researchers who commit to acting as stewards of underrepresented trainees.

#### Research governance and team structure

In our research, rarely was governance central to strategies to include underrepresented team members into organizational decision-making. Whereas our interviewees highlighted the need for WFD because it was central to their goals for greater inclusions in PMR, whether and how diverse team members augment the value of their studies beyond their contributions to the recruitment and retention of diverse research participants remained elusive. Indeed, frontline staff workers felt their contributions elsewhere were undervalued, and often not sought at all. The concentration of underrepresented team members in such “diversity work” exclusively suggests structural inequities in the research enterprise.

## Limitations

While our study provides in-depth analysis of five studies across three PMR consortia, other studies and consortia may more intentionally address WFD concerns. However, because our data come from studies with diversity mandates of research participation, our results likely reflect common practices across PMR initiatives where explicit WFD initiatives are not yet underway.

## Conclusion

Our study demonstrates a need for increasing diversity on research teams through commitments to equity and structural reform. Past efforts to increase diversity in the genomics and PMR workforce have often been instrumental, unintentionally deepening inequities within the research ecosystem. The responsibility to create equity in WFD falls on the shoulders of all who participate in scientific discovery—particularly those who have benefited from privilege and status in these spaces. Equitable WFD will only be achieved when structural conditions ensure underrepresented researchers are prioritized and supported to thrive.

Equitable diversification calls for new centers of power within study teams and the broader research ecosystem. Recognition of existing hierarchies and power structures in the research ecosystem compound challenges for equitable diversification. Tokenism and instrumental diversity jeopardize goals to diversify research teams and risk merely transient and superficial diversification. The siloing of expertise of underrepresented team members to frontline and diversity-only activities may also perpetuate a turnstile effect, in which these team members move from study to study laterally, or altogether leave the field because of the lack of advancement opportunities. Without taking into account an ecosystem framework that addresses the conditions that structure power within research teams, tokenism can be misrecognized as inclusion.

## References

[1] American Society of Human Genetics, 2020. https://www.ashg.org/about/diversity-inclusion-policy/

[2] National Society of Genetic Counselors, 2020. https://www.nsgc.org/page/diversity,-equity-and-inclusion-resources

[3] National Human Genome Research Insititute. *Building a Diverse Genomics Workforce: An NHGRI Action Agenda*. 2020.

[4] Bonham VL, Green ED. The genomics workforce must become more diverse: a strategic imperative. The American Journal of Human Genetics. 2021;108(1):3–7. doi: 10.1016/j.ajhg.2020.12.013 33417888 PMC7820786

[5] National Institutes of Health, 2021. https://www.nih.gov/ending-structural-racism/unite

[6] Lee SS-J, Fullerton SM, Saperstein A, Shim JK. Ethics of inclusion: cultivate trust in precision medicine. Science. 2019;364(6444):941–942. doi: 10.1126/science.aaw8299 31171685 PMC7261394

[7] Hofstra B, Kulkarni VV, Galvez SM-N, He B, Jurafsky D, McFarland DA. The diversity–innovation paradox in science. Proceedings of the National Academy of Sciences. 2020;117(17):9284–9291. 10.1073/pnas.1915378117 PMC7196824 32291335

[8] AlShebli BK, Rahwan T, Woon WL. The preeminence of ethnic diversity in scientific collaboration. Nature communications. 2018;9(1):1–10. doi: 10.1038/s41467-017-02088-w 30514841 PMC6279741

[9] Green ED, Gunter C, Biesecker LG, et al. Strategic vision for improving human health at The Forefront of Genomics. Nature. 2020;586(7831):683–692. doi: 10.1038/s41586-020-2817-4 33116284 PMC7869889

[10] Collins F.S., Adams A.B., Aklin C., Archer T.K., Bernard M.A., Boone E., et al. 2021. Affirming NIH’s commitment to addressing structural racism in the biomedical research enterprise. Cell, 184(12), pp.3075–3079. doi: 10.1016/j.cell.2021.05.014 34115967

[11] Braveman P. What are health disparities and health equity? We need to be clear. Public health reports. 2014;129(1_suppl2):5–8. doi: 10.1177/003335491412900102 24385658 PMC3863701

[12] Braveman P, Gruskin S. Defining equity in health. J Epidemiol Community Health. 2003;57(4):254. doi: 10.1136/jech.57.4.254 12646539 PMC1732430

[13] Hoppe TA, Litovitz A, Willis KA, et al. Topic choice contributes to the lower rate of NIH awards to African-American/black scientists. Science Advances. 2019;5(10):eaaw7238. doi: 10.1126/sciadv.aaw7238 31633016 PMC6785250

[14] Jimenez MF, Laverty TM, Bombaci SP, Wilkins K, Bennett DE, Pejchar L. Underrepresented faculty play a disproportionate role in advancing diversity and inclusion. Nature ecology & evolution. 2019;3(7):1030–1033. doi: 10.1038/s41559-019-0911-5 31160738

[15] Eaton AA, Saunders JF, Jacobson RK, West K. How gender and race stereotypes impact the advancement of scholars in STEM: Professors’ biased evaluations of physics and biology post-doctoral candidates. Sex Roles. 2020;82(3):127–141.

[16] Thomson RA Jr, Salazar ES, Howard Ecklund E. The very ivory tower: pathways reproducing racial-ethnic stratification in US academic science. Ethnic and Racial Studies. 2020:1–21.

[17] Barber PH, Hayes TB, Johnson TL, Márquez-Magaña L. Systemic racism in higher education. Science. 2020;369(6510):1440–1441. doi: 10.1126/science.abd7140 32943517

[18] Stevens KR, Masters KS, Imoukhuede PI, et al. Fund black scientists. Cell. 2021. doi: 10.1016/j.cell.2021.01.011 33503447

[19] Charmaz K. Constructing grounded theory. Sage; 2014.

[20] Fryer CS, Passmore SR, Maietta RC, et al. The symbolic value and limitations of racial concordance in minority research engagement. Qualitative health research. 2016;26(6):830–841. doi: 10.1177/1049732315575708 25769299 PMC4658313

[21] Flores GM. Racialized tokens: Latina teachers negotiating, surviving and thriving in a white woman’s profession. Qualitative Sociology. 2011;34(2):313–335.

[22] Jackson PB, Thoits PA, Taylor HF. Composition of the workplace and psychological well-being: The effects of tokenism on America’s Black elite. Social Forces. 1995;74(2):543–557.

[23] Smith-Doerr L, Alegria SN, Sacco T. How diversity matters in the US science and engineering workforce: A critical review considering integration in teams, fields, and organizational contexts. Engaging Science, Technology, and Society. 2017;3:139–153.

[24] Olsen LD. The conscripted curriculum and the reproduction of racial inequalities in contemporary US medical education. Journal of health and social behavior. 2019;60(1):55–68. doi: 10.1177/0022146518821388 30650990

[25] Puritty C, Strickland LR, Alia E, et al. Without inclusion, diversity initiatives may not be enough. Science. 2017;357(6356):1101–1102. doi: 10.1126/science.aai9054 28912234

[26] Dzirasa K. Revising the a Priori Hypothesis: Systemic Racism Has Penetrated Scientific Funding. Cell. 2020;183(3):576–579. doi: 10.1016/j.cell.2020.09.026 33125883

[27] Heggeness ML, Gunsalus KTW, Pacas J, McDowell G. The new face of US science. Nature News. 2017;541(7635):21. doi: 10.1038/541021a 28054625

[28] New NIH Approach to Grant Funding Aimed at Optimizing Stewardship of Taxpayer Dollars [press release]. 2017.

[29] Ginther DK, Schaffer WT, Schnell J, et al. Race, ethnicity, and NIH research awards. Science. 2011;333(6045):1015–1019. doi: 10.1126/science.1196783 21852498 PMC3412416

[30] Lee SS-J, Vasquez EE, Jeske M, et al. Precision in practice: Negotiating diversity and inclusion in precision medicine research. Social Studies of Science. Virtual, 2020.

[31] Gibbs Jr KD, Griffin KA. What do I want to be with my PhD? The roles of personal values and structural dynamics in shaping the career interests of recent biomedical science PhD graduates. CBE—Life Sciences Education. 2013;12(4):711–723. 10.1187/cbe.13-02-0021 PMC3846521 24297297

[32] Gibbs KD Jr, McGready J, Bennett JC, Griffin K. Biomedical science Ph. D. career interest patterns by race/ethnicity and gender. PLoS ONE. 2014;9(12):e114736. doi: 10.1371/journal.pone.0114736 25493425 PMC4262437

